# Associated factors in SARS-CoV-2 infection among close contacts during the zero-COVID policy from 2020 to 2022 in the northeast of Shenzhen, China: a retrospective cohort study

**DOI:** 10.3389/fpubh.2025.1589683

**Published:** 2025-06-11

**Authors:** Yu Zeng, Jiaqi Xv, Li Cui

**Affiliations:** Longgang Center for Disease Control and Prevention in Shenzhen, Shenzhen, China

**Keywords:** close contacts, SARS-CoV-2, associated factors, vaccination, secondary attack rate

## Abstract

**Background:**

Identifying high-risk groups and developing specific interventions is essential to combat pandemic including COVID-19. We estimated key factors of demographic characteristic, exposure-related variables, and vaccination status in secondary infection among close contacts throughout the zero-COVID policy.

**Methods:**

We used contact tracing data from 622 primary cases and 31,278 close contacts between February 2020 and December 2022 in the northeast of Shenzhen, China. The multivariate logistic regression was utilized to identify factors affecting SARS-CoV-2 infection of close contacts.

**Results:**

The secondary attack rate (SAR) of close contacts was 1.4% (95% CI: 1.3–1.5%), and the associated factors included over 50 (OR = 1.47, 95% CI: 1.13–1.91), living in urban village housing (OR = 1.51, 95% CI: 1.04–2.19), closer relationship with primary cases (e.g., household members: OR = 44.06, 95% CI: 34.45–56.36), last exposure occurring before the illness onset of cases (e.g., >2 days before: OR = 0.52, 95% CI: 0.27–1.00), exposed to cases with moderate symptoms (OR = 0.62, 95% CI: 0.40–0.95), and better COVID-19 vaccination status (e.g., booster vaccination within 6 months before the last exposure: OR = 0.38, 95% CI: 0.24–0.60).

**Conclusion:**

Our findings should be helpful to develop targeted surveillance and interventions for these high-risk groups to understand ongoing COVID-19 issue and improve future pandemic management.

## 1 Introduction

Since December 2019, the coronavirus disease 2019 (COVID-19) caused by severe acute respiratory syndrome coronavirus 2 (SARS-CoV-2) has rapidly progressed into a global pandemic, resulting in major economic and social repercussions worldwide (1–3). By December 7, 2022, the end of the dynamic zero-COVID policy in China, over 642.38 million SARS-CoV-2 cases and 6.62 million deaths has been reported from more than 200 countries and regions (4).

Contact tracing has been empirically confirmed as an effective public health response to the containment of SARS-CoV-2 transmission in the implementation of zero-COVID policy for nearly 3 years (5, 6), just as it worked for many emerging infectious diseases including Ebola virus disease (7), severe acute respiratory syndrome (SARS) (8), and Middle East respiratory syndrome (MERS) (9). Close contacts, those who had potentially risky encounter with SARS-CoV-2 cases during the period of pathogen transmission, are the critical group at the highest infection risk (10). Plenty of studies attempted to examine factors affecting SARS-CoV-2 acquisition among close contacts with a view to further identifying potentially infected individuals and preventing the onward transmission of COVID-19.

However, existing recorded demographic and exposure-related factors vary widely, limiting our understanding of key risks and specific confounders in the spread of COVID-19 and the development trend of the pandemic (11, 12). Meanwhile, since the vast majority of previous analyses relied on limited contact tracing data of early 2020 (11–13), while the mass vaccination effort in China did not launch until the end of that year (14), there is a dearth of studies on the specific impact of COVID-19 vaccination on the vulnerability of infection. It is also unknown whether the risk factors identified in the early stage of pandemic will continue to influence infections throughout the implementation of zero-COVID policy, independent of varying circulating variants and response strategies in subsequent waves. Such insights should be crucial to formulate scientific measures to prevent and contain airborne epidemics at present and in the future.

To identify key factors of demographic characteristic, exposure-related variables, and vaccination status contributing to secondary infection among close contacts and improve response strategies for ongoing COVID-19 health issue and future public health emergency, we undertook a retrospective cohort study of associated factors in SARS-CoV-2 seropositivity in the northeast of Shenzhen throughout the zero-COVID policy from 2020 to 2022.

## 2 Methods

### 2.1 Study area

Shenzhen is a coastal city with a permanent population of over 17 million in Southern China, serving as a major international financial center and transportation hub. Among the 10 administrative districts of Shenzhen, Longgang district is the second biggest in terms of area and population, and the city’s sole hospital designated for SARS-CoV-2 treatment is also located here.

### 2.2 Study setting

This was a retrospective cohort study to evaluate the associated factors in secondary infection of SARS-CoV-2 among close contacts in the northeast of Shenzhen (Longgang district), China. The study included close contacts of local SARS-CoV-2 cases identified from February 3, 2020 (i.e., the date of the first local case reported), to December 7, 2022 (i.e., the end of the zero-COVID policy).

### 2.3 Definitions

Local SARS-CoV-2 cases referred to residents with permanent address in Longgang district who were confirmed by positive quantitative real-time polymerase chain reaction (RT-qPCR) testing.

Close contacts were usually defined as individuals interacted with SARS-CoV-2 cases without effective protection in short range, starting 2 days before the onset of illness (i.e., clinical symptoms for confirmed cases or nucleic acid sampling for the asymptomatic) (15). This definition varied slightly according to distinct policy regarding different risk of transmission. For instance, the identification period for the interaction was once extended to 4 days before the illness onset for the surge in cases due to Delta variant (12). After identification, close contacts were typically quarantined in a central facility or at home for 5–21 days from the last exposure to a primary case, depending on specific policy. During the quarantine, regular RT-qPCR testing would be conducted invariably. The release of close contacts was contingent upon persistently negative results throughout the isolation, while those once tested positive were classified as secondary cases.

Vaccination status was categorized under the national technical recommendations for COVID-19 vaccination as follow: full vaccination if individuals received one dose of adenovirus vector vaccine, two doses of inactivated vaccine, or three doses of recombinant subunit vaccine; partial vaccination if received one dose of inactivated vaccine, or one to two doses of recombinant subunit vaccine; and booster vaccination if received two doses of adenovirus vector vaccine, or one dose of any aforementioned types of vaccines (i.e., adenovirus vector vaccine, inactivated vaccine, and recombinant subunit vaccine) following the first two doses of inactivated vaccine (16, 17). As the COVID-19 vaccine effectiveness against SARS-CoV-2 infections decreased by 6 months after inoculation, the above vaccination status was further divided according to whether the interval between last inoculation and last exposure exceeded 6 months (18–20).

### 2.4 Data collection

All information regarding close contacts was obtained through filed epidemiological investigation from the Center for Disease Control and Prevention (CDC) of Longgang, Shenzhen. This contact tracing dataset documented comprehensive information, including demographic characteristics, specifics of exposure to SARS-CoV-2 cases, and COVID-19 vaccination status, such as gender, age, nationality, occupation, relationship with the primary case, date of the last exposure, clinical severity of primary case, dose, type, and manufacturer of vaccine, time of each inoculation.

### 2.5 Ethical approval

As a component of the public health response to COVID-19 issued by the National Health Commission of China, data collection here was exempt from institutional review board and the informed consent was also waived (10). Ethical approval for the data analysis was provided by the ethics committee of Longgang CDC (No. LGCDC2022006).

### 2.6 Statistical analysis

Frequencies and percentages were performed to describe categorical variables, while median and interquartile range (IQR) for continuous variables of non-normal distribution. The secondary attack rate (SAR) was used to estimate the onward transmission risk of SARS-CoV-2, which was calculated as the proportion of secondary cases among the total number of close contacts. The 95% confidence intervals (95%CI) of SAR was calculated using interval estimation for binomial proportion (21). Logistic regression models were used to estimate the association between factors of interest and secondary infection of SARS-CoV-2 among close contacts by calculating odds ratio (OR). Demographic characteristics (gender, age, and type of housing), exposure factors (relationship with primary cases, clinical severity of primary cases, time of last exposure), and vaccination status were included into models as predictors. Univariate logistic regression models were fitted for each variable independently and select those with *p-*value < 0.15 for a multivariate analysis. The variance inflation factor (VIF) was calculated to assess collinearity for variables in the multivariate model, and no collinearity was detected since all VIF values was less than 10. A two-sided statistical hypothesis test with *p*-value < 0.05 was considered statistically significant. All data analyses were conducted using R software (version 4.3.2).

## 3 Results

**Figure 1:**
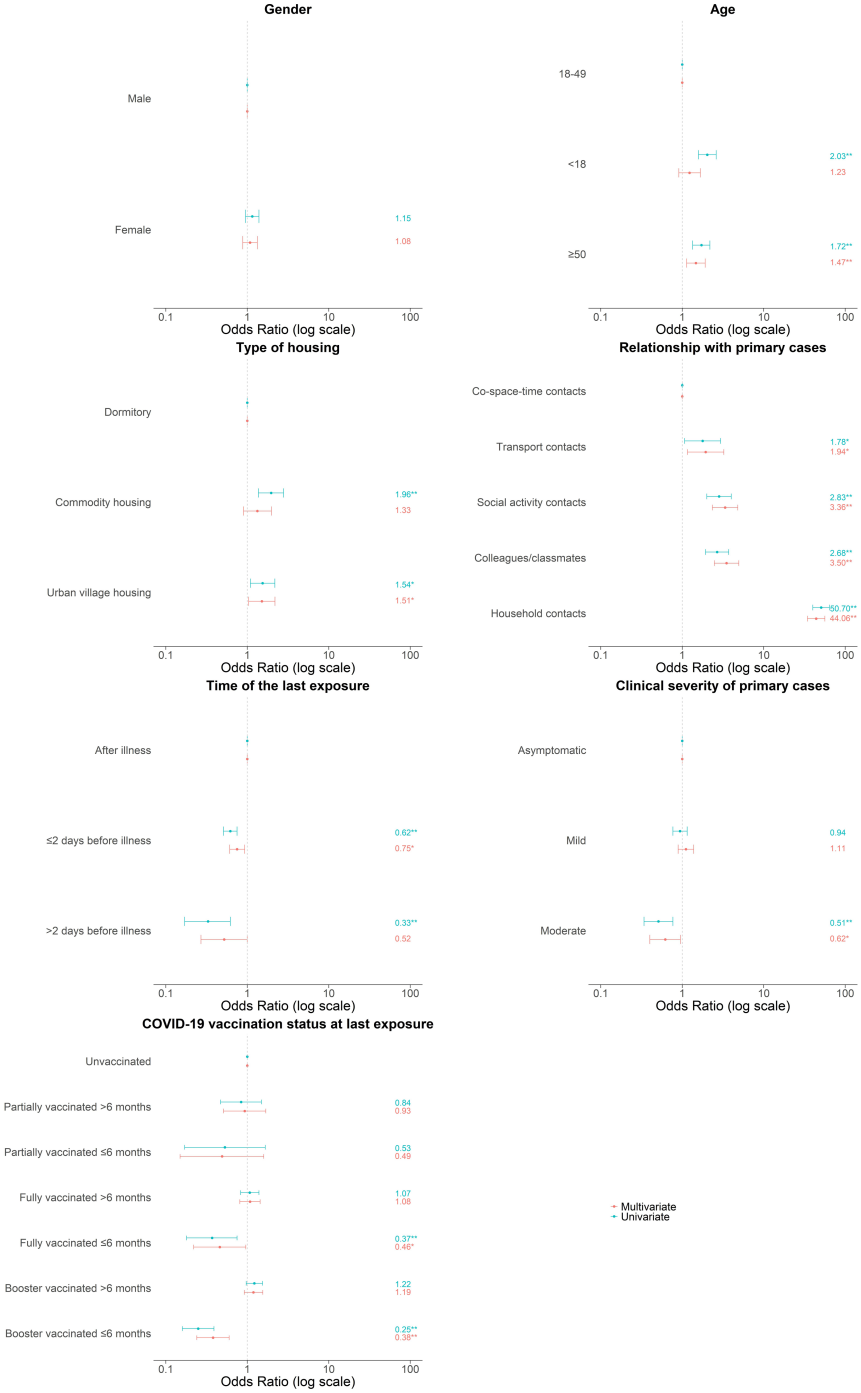
Associated factors in secondary infection of SARS-CoV-2 among close contacts. Odds ratio were indicated by points and 95% confidence interval were indicated by error bars. *p < 0.05; **p < 0.01.

From February 3, 2020, to December 7, 2022, 622 local SARS-CoV-2 cases in Longgang district of Shenzhen were reported, with 60.0% (373/622) symptomatic and 40.0% (249/622) asymptomatic, including 1 death. Meanwhile, 31,278 close contacts of 622 primary cases were identified, with a median of 6 contacts per case (IQR: 2–40). As shown in Table 1, the majority of the close contacts were male (53.0%, 16,581/31278), with a median age of 32 years (IQR: 24–43). Approximately one-seventieth (1.4, 95%CI: 1.3–1.5%) close contacts were identified as secondary cases, among whom 57.6% (249/432) were symptomatic and 42.4% (183/432) asymptomatic. Six of seven variables encompassing demographic characteristics, exposure factors, and vaccination status had significant impact on SARS-CoV-2 infection in multivariate logistic regression model (Figure 1).

**Table 1:** Secondary attack rate (SAR) of SARS-CoV-2 among close contacts from 2020 to 2022 in the northeast of Shenzhen, China (*N* = 31,278).

Variables	All, *N* (%)	Infection, *N* (%)	SAR (95%CI)
Gender			
Male	16,581 (53.0)	214 (49.5)	1.3 (1.1–1.5)
Female	14,697 (47.0)	218 (50.5)	1.5 (1.3–1.7)
Age, years			
18–49	23,230 (74.3)	264 (61.1)	1.1 (1.0–1.3)
<18	3,550 (11.3)	81 (18.8)	2.3 (1.8–2.8)
≥50	4,498 (14.4)	87 (20.1)	1.9 (1.5–2.3)
Type of housing			
Dormitory	4,364 (14.0)	38 (8.8)	0.9 (0.6–1.1)
Commodity housing	9,652 (30.9)	163 (37.7)	1.7 (1.4–1.9)
Urban village housing	17,262 (55.2)	231 (53.5)	1.3 (1.2–1.5)
Relationship with primary cases			
Sharing the same space and time	22,419 (71.7)	125 (28.9)	0.6 (0.5–0.7)
Taking the same transport	1721 (5.5)	17 (3.9)	1.0 (0.5–1.5)
Having social interaction	2,815 (9.0)	44 (10.2)	1.6 (1.1–2.0)
Colleagues or classmates	3,442 (11.0)	51 (11.8)	1.5 (1.1–1.9)
Roommates or household members	881 (2.8)	195 (45.1)	22.1 (19.4–24.9)
Time of the last exposure^a^			
After the case’s onset of illness	9,815 (31.4)	187 (43.3)	1.9 (1.6–2.2)
≤2 days before the onset of illness	19,891 (63.6)	235 (54.4)	1.2 (1.0–1.3)
>2 days before the onset of illness	1,572 (5.0)	10 (2.3)	0.6 (0.2–1.0)
Clinical severity of primary cases^b^			
Asymptomatic	9,999 (32.0)	152 (35.2)	1.5 (1.3–1.8)
Mild	17,594 (56.3)	251 (58.1)	1.4 (1.3–1.6)
Moderate	3,685 (11.8)	29 (6.7)	0.8 (0.5–1.1)
COVID-19 vaccination status at last exposure			
Unvaccinated	9,381 (30.0)	145 (33.6)	1.5 (1.3–1.8)
Partially vaccinated for over 6 months	998 (3.2)	13 (3.0)	1.3 (0.6–2.0)
Partially vaccinated for less than 6 months	365 (1.2)	3 (0.7)	0.8 (0.3–2.4)
Fully vaccinated for over 6 months	5,914 (18.9)	98 (22.7)	1.7 (1.3–2.0)
Fully vaccinated for less than 6 months	1,403 (4.5)	8 (1.9)	0.6 (0.2–1.0)
Booster vaccinated for over 6 months	7,590 (24.3)	143 (33.1)	1.9 (1.6–2.2)
Booster vaccinated for less than 6 months	5,627 (18.0)	22 (5.1)	0.4 (0.2–0.6)

CI, confidence interval; NA, not applicable.

^a^The onset of illness referred to the beginning of clinical symptoms for confirmed cases or the date of nucleic acid sampling among the asymptomatic.

^b^As only seven close contacts were exposed to primary cases with severe symptoms, they were merged into “Moderate”.

### 3.1 Demographic characteristic in secondary infection

As shown in Table 2, no gender difference in SARS-CoV-2 seropositivity of close contacts was found. Notably, the SAR of close contacts under 18 (2.3%) or over 50 (1.9%) was higher than that of those aged 18–49 (1.1%), and the difference was also demonstrated in univariate model. However, the link between under-18 contacts and increased infection risk was no longer significant, while those over 50 were still had higher risk in multivariate analysis (OR = 1.47, 95%CI: 1.13–1.91). Close contacts living in urban village housing (1.3%) and commodity housing (1.7%) had higher SAR than residents in dormitory (0.9%), being consistent with the trend in a univariate model. However, only living in urban village housing was still related to increased risk of infection in multivariate model (OR = 1.51, 95%CI: 1.04–2.19).

**Table 2:** Demographic characteristic in secondary infection of SARS-CoV-2 among close contacts.

Demographic characteristic	SAR (95%CI)	Odds Ratio (95% CI)	
		Univariate	Multivariate
Gender			
Male	1.3 (1.1–1.5)	1[reference]	1[reference]
Female	1.5 (1.3–1.7)	1.15 (0.95–1.39)	1.08 (0.88–1.33)
Age, years			
18–49	1.1 (1.0–1.3)	1[reference]	1[reference]
<18	2.3 (1.8–2.8)	2.03 (1.58–2.61)	1.23 (0.91–1.67)
≥50	1.9 (1.5–2.3)	1.72 (1.34–2.19)	1.47 (1.13–1.91)
Type of housing			
Dormitory	0.9 (0.6–1.1)	1[reference]	1[reference]
Commodity housing	1.7 (1.4–1.9)	1.96 (1.37–2.79)	1.33 (0.90–1.98)
Urban village housing	1.3 (1.2–1.5)	1.54 (1.09–2.18)	1.51 (1.04–2.19)

### 3.2 SARS-CoV-2 exposure factors in secondary infection

As shown in Table 3, the predominant relationship between close contacts and primary cases were strangers sharing the same space and time (71.7%, 22,419/31278), followed by colleagues or classmates (11.0%, 3442/31278), people in social interaction (9.0%, 2815/31278), people in the same transport (5.5%, 1721/31278), and household members or roommates (2.8%, 881/31278). Compared to close contacts only in co-space–time interaction with primary cases, others sharing more closer relationships had much higher SAR (0.6% versus 3.6%). The significantly increased infection risk among those living with (OR = 44.06, 95% CI: 34.45–56.36), working or learning with (OR = 3.50, 95% CI: 2.48–4.94), socially interacting with (OR = 3.36, 95% CI: 2.34–4.81), and taking the same transport with primary cases (OR = 1.94, 95% CI: 1.16–3.24) were likewise indicated in multivariate analysis.

**Table 3:** SARS-CoV-2 exposure factors in secondary infection of SARS-CoV-2 among close contacts.

SARS-CoV-2 exposure factors	SAR (95%CI)	Odds Ratio (95% CI)	
		Univariate	Multivariate
Relationship with primary cases			
Sharing the same space and time	0.6 (0.5–0.7)	1[reference]	1[reference]
Taking the same transport	1.0 (0.5–1.5)	1.78 (1.07–2.96)	1.94 (1.16–3.24)
Having social interaction	1.6 (1.1–2.0)	2.83 (2.00–4.00)	3.36 (2.34–4.81)
Colleagues or classmates	1.5 (1.1–1.9)	2.68 (1.93–3.72)	3.50 (2.48–4.94)
Roommates or household members	22.1 (19.4–24.9)	50.70 (40.00–64.26)	44.06 (34.45–56.36)
Time of the last exposure			
After the case’s onset of illness	1.9 (1.6–2.2)	1[reference]	1[reference]
≤2 days before the onset of illness	1.2 (1.0–1.3)	0.62 (0.51–0.75)	0.75 (0.61–0.93)
>2 days before the onset of illness	0.6 (0.2–1.0)	0.33 (0.17–0.62)	0.52 (0.27–1.00)
Clinical severity of primary cases			
Asymptomatic	1.5 (1.3–1.8)	1[reference]	1[reference]
Mild	1.4 (1.3–1.6)	0.94 (0.77–1.15)	1.11 (0.89–1.38)
Moderate	0.8 (0.5–1.1)	0.52 (0.34–0.77)	0.62 (0.40–0.95)

Approximately two-thirds (68.6%, 19,891/31278) of the last exposure occurred within 2 days prior to the onset of illness in primary cases, with the remainder distributed between after that (31.4%, 9815/31278) and over 2 days before (5.0%, 1572/31278). Relative to close contacts exposed after the illness onset (1.9%), those exposed within 2 days before had decreased SAR (1.2%), let alone those exposed over 2 days before (0.6%). In multivariate analysis, last exposure within 2 days before the illness onset (OR = 0.75, 95% CI: 0.61–0.93) and over 2 days before (OR = 0.52, 95% CI: 0.27–1.00) were both correlated with lower infection risk.

More than half of close contacts were exposed to primary cases with mild symptoms (56.3%, 17,594/31278), followed by the asymptomatic (32.0%, 9999/31278), and those with moderate symptoms (11.8%, 3685/31278). In comparison to close contacts of the asymptomatic, those of moderate cases showed a decreased SAR (1.5% versus 0.8%), while contacts of mild cases had a approximately equal SAR (1.5% versus 1.4%). Similarly, only close contacts exposed to moderate cases were less likely to infect SARS-CoV-2 (OR = 0.62, 95% CI: 0.40–0.95) in multivariate analysis.

### 3.3 COVID-19 vaccination status and secondary infection

In total, 70.0% (21,897/31278) of close contacts had been immunized with at least one dose of COVID-19 vaccine before their last exposure to the primary cases, among which the most prevalent status was booster vaccination, followed by full vaccination and partial vaccination. The overall SAR of the vaccinated contacts was slightly lower than the unvaccinated (1.3% versus 1.5%). Multivariate analysis also revealed that close contacts who received booster dose within 6 months before last exposure had the lowest infection risk (OR = 0.38, 95% CI: 0.24–0.60), and then the fully vaccinated within 6 months before (OR = 0.46, 95% CI: 0.22–0.96). Nevertheless, neither the booster (OR = 1.19, 95% CI: 0.92–1.55) nor full vaccination (OR = 1.08, 95% CI: 0.81–1.44) given over 6 months prior to the last exposure were associated with a reduced infection risk. Furthermore, infection risks were similar for partially vaccinated and unvaccinated contacts, regardless of the timing of inoculation (≤6 months: OR = 0.49, 95% CI: 0.15–1.59; >6 months: OR = 0.93, 95% CI: 0.51–1.69) (Table 4).

**Table 4:** Vaccination status in secondary infection of SARS-CoV-2 among close contacts.

COVID-19 vaccination status at last exposure	SAR (95%CI)	Odds Ratio (95% CI)	
		Univariate	Multivariate
Unvaccinated	1.5 (1.3–1.8)	1[reference]	1[reference]
Partially vaccinated for over 6 months	1.3 (0.6–2.0)	0.84 (0.47–1.49)	0.93 (0.51–1.69)
Partially vaccinated for less than 6 months	0.8 (0.3–2.4)	0.53 (0.17–1.66)	0.49 (0.15–1.59)
Fully vaccinated for over 6 months	1.7 (1.3–2.0)	1.07 (0.83–1.39)	1.08 (0.81–1.44)
Fully vaccinated for less than 6 months	0.6 (0.2–1.0)	0.37 (0.18–0.75)	0.46 (0.22–0.96)
Booster vaccinated for over 6 months	1.9 (1.6–2.2)	1.22 (0.97–1.54)	1.19 (0.92–1.55)
Booster vaccinated for less than 6 months	0.4 (0.2–0.6)	0.25 (0.16–0.39)	0.38 (0.24–0.60)

## 4 Discussion

To date, this is one of the most comprehensive analysis of SARS-CoV-2 infection among close contacts across the course of zero-COVID policy implementation from 2020 to 2022. Our study found an overall SAR among close contacts of 1.4%, much lower than 18.4% reported in a previous study, while the specific SAR of household contacts were comparable (22.1% versus 23.3%) (22, 23). In addiction to the possibility of difference in population density and pathogen variants, it was probably related to the stricter prevention measures. Specifically, the average of close contacts traced from each local COVID-19 cases in our study was 50.3 (31,278/622), being over four times than the previous estimate of 11.2 (87,838/7818) in a systematic review (12). Therefore, public health authorities need to balance the benefits of infection control and the huge burden created by stringent contact tracing when facing future outbreak. Further identification of high-risk groups in close contact by exploring associated factors in secondary infection, is a practical approach to promote precise contact tracing and reduce surveillance burden.

Consistent with the existing research, the highest infection risk among household contacts was also observed in our study (24, 25). Given the predominant modes of transmission for SARS-CoV-2 of respiratory droplets and close contact (26), daily interactions without masks in short distance among individuals living together naturally form the transmission characteristics of household clustering (27, 28). It is worth emphasizing that the role of close contacts outside the household as the susceptible population were also sufficiently studied here, where colleagues or classmates had the second highest risk, followed by social activity contacts, transportation contacts, and co-space–time contacts. These findings indicate that the closer the relationship with primary cases, the higher the risk of infection, which is helpful to guide the prioritization of contact tracing. That is, household contacts should be given priority to identify and monitor, while co-space–time contacts the last to consider. At the individual levels, consistently wearing masks and enhancing indoor ventilation play crucial roles in reducing transmission, particularly within household environments.

Last exposure occurred before the onset of illness was linked with reduced infection risk, also being in line with prior findings (13). It’s probably due to lower contagiousness in the incubation period, the stage before PCR-positive of the asymptomatic included (29). In preceding observations of naturally infected individuals, the viral loads of SARS-CoV-2 peaked in the early symptomatic period (29–31). An artificial infection trial also confirmed this finding by reporting that peak viral loads were reached 4–5 days after inoculation, later than the onset of symptoms appeared 2–4 days after inoculation (32). In view of the trend reflected from our findings of the longer the interval between last exposure of contacts and illness onset of cases, the lower the infection risk, the contact tracing strategy should be optimized based on this interval. That is, monitoring contacts in post-symptomatic exposure or PCR-positive period as priority, then those exposed within 2 days before illness, and lastly those exposed over 2 days before.

There were conflicting findings regarding the role of age in SARS-CoV-2 seropositivity among close contacts. One systematic review found higher infection rates in the 'older adult (12), whereas another meta-analysis reported no significant impact of aging on infection risk (11). In our study, close contacts aged over 50 were more likely to acquire SARS-CoV-2. Age-related decline and impairment of immune are considered to be the major reasons for the increased susceptibility to respiratory infections such as SARS-CoV-2 in old people (33). Thus, more targeted prevention measures should be developed to protect old age from infection and related adverse health outcomes during the pandemic, such as more information about health promotion delivered through age-friendly channels.

Contrary to the popular belief that exposure to symptomatic cases constituted higher infection risk of SARS-CoV-2 (12), we found a lower risk in close contacts exposed to cases with moderate symptoms, whereas that was comparable between those exposed to mild and asymptomatic cases. Considering no consensus on whether symptomatic cases have a higher viral shedding than asymptomatic, the presence of clinical manifestations is not a reliable indicator of increased infectiousness (34). Our finding may be attributed to the possibility that less virus was effectively transmitted from moderate cases, as both the susceptible and the infected were prone to adopt stricter protection measures in the face of apparent symptoms. However, further exploration is needed to offer support, especially those focusing changing risk factors for developing SARS-CoV-2 infection among different variants (35).

Several unique factors related to SARS-CoV-2 infection in close contacts were also identified, one of which was COVID-19 vaccination status. The booster vaccinated within 6 months prior to the last exposure had the lowest risk, with the fully vaccinated during that also demonstrating reduced risk. However, once the immunization lasted for over 6 months, these association were no longer significant. Additionally, no differences in the susceptibility to infection were found between partially vaccinated and unvaccinated contacts. These findings can be ascribed to the COVID-19 vaccines efficacy against infection, with booster and full vaccination being more effective than partial vaccination (36). Although the exact duration of protection from SARS-CoV-2 infection generated following immunization is laborious to evaluate and is likely to vary between individuals, a notable decrease in antibodies levels has been observed with time (20, 37). Our study also provided empirical evidence that the protection against infection did decay somewhat 6 months after booster or full vaccination, which aligns with global evidence (38, 39). Therefore, what counts is to timely complete the recommended vaccine series and receive booster dose to ensure optimal protection. Meanwhile, the importance of consistently taking personal preventive measures cannot be overlooked, especially the unvaccinated, partially vaccinated, and those in full or booster vaccination for over 6 months.

Residing in urban village housing was identified as an unique risk factor as well, likely because of the complex population composition and diverse spatial layout. Unlike dormitories typically enclosed within collective units and commodity housing separated from commercial areas by gardens, urban village housing blends residential and commercial zones, where this convenience may lead to more frequent contact between residents and potential infected individuals (40). Moreover, the overcrowded condition in urban village is a well-documented risk for the spread of infectious diseases, particularly the respiratory ones (41). The poor indoor ventilation conditions might also lead to an increase in viral load (42–44). This finding underscores the urgent need for implementing more stringent non-pharmaceutical interventions to improve air environment of both indoor and in the public area of urban villages during the pandemic, and promoting health education among residents to shield them from respiratory infection like COVID-19. In the long run, developers are suppose to provide more space to residents at an acceptable price to avoid the excessive congestion of indoor space or excessive compression of public activity space.

This study has some limitations. First, inconsistency or noise in definitions may arise due to the involvement of multiple investigation teams in data collection and the inevitable updates of epidemiological survey protocols when the situation changed dramatically. However, such variation is nearly unavoidable in any active outbreak response. Second, despite utilizing a large dataset of over 30,000 close contacts, the sample size of some subsets was limited, potentially resulting in insufficient power to detect statistical significance. Third, the lack of evaluation in different phases of the pandemic divided by specific circulating variants and preventive measures, may limited the understanding of nuanced variation in factors related to secondary infection over time.

In summary, the older adult over 50, residents in urban village housing, those in closer relationship with cases (e.g., household contacts), exposed after the illness onset of cases, exposed to asymptomatic cases, and in poor vaccination status (i.e., the unvaccinated, partially vaccinated, and fully or booster vaccinated for over 6 months) were more vulnerable to SARS-CoV-2 infection. To better understand the ongoing COVID-19 health issue and improve preparedness strategies for similar public health emergencies in the future, targeted surveillance and interventions should give priority to these high-risk groups.

## Data Availability

Data used for conducting this research is unavailable due to privacy or ethical restrictions. Requests to access the datasets should be directed to Yu Zeng, zengy56@mail2.sysu.edu.cn.
