# Supplementary material for: Associated factors in SARS-CoV-2 infection among close contacts during the zero-COVID policy from 2020 to 2022 in the northeast of Shenzhen, China: a retrospective cohort study
Source: Front Public Health. 2025 Jun 11;13:1589683. doi: 10.3389/fpubh.2025.1589683 (PMC12187859; doi:10.3389/fpubh.2025.1589683)
Supplement: Supplementary file 1 [file Data_Sheet_1.pdf]

Supplementary materials（Manuscript ID :1589683）

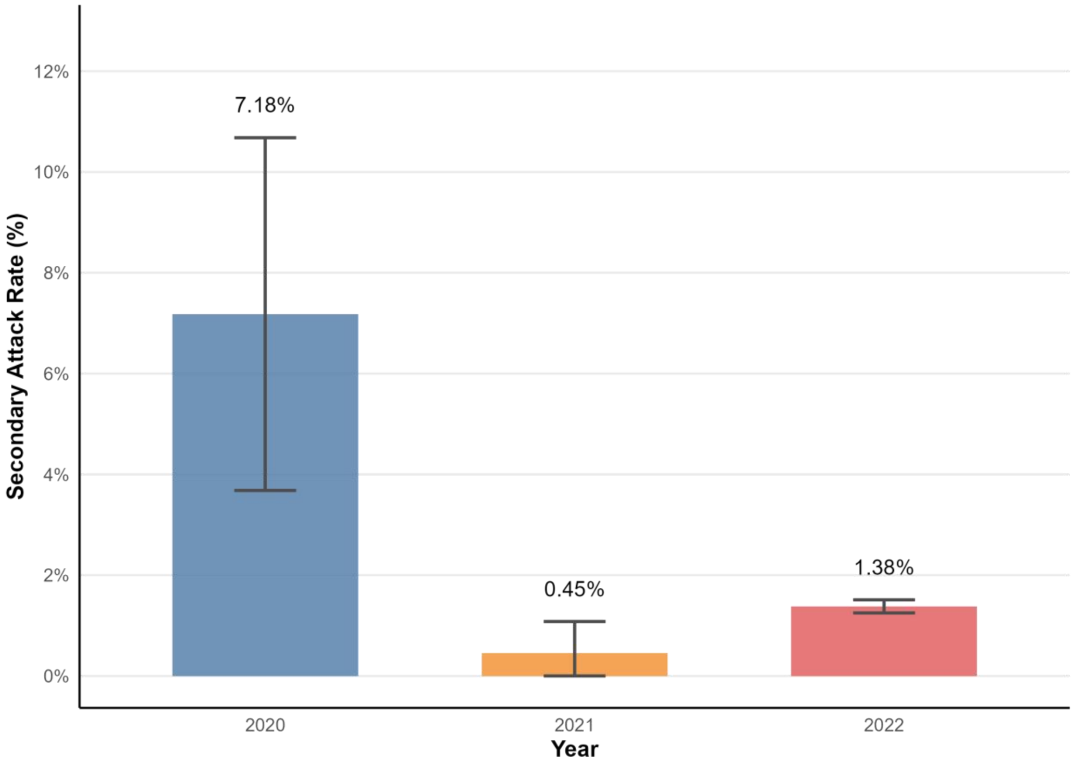

Supplementary Figure1. The secondary attack rates of SARS-CoV-2 among close contacts during 2020-2022.

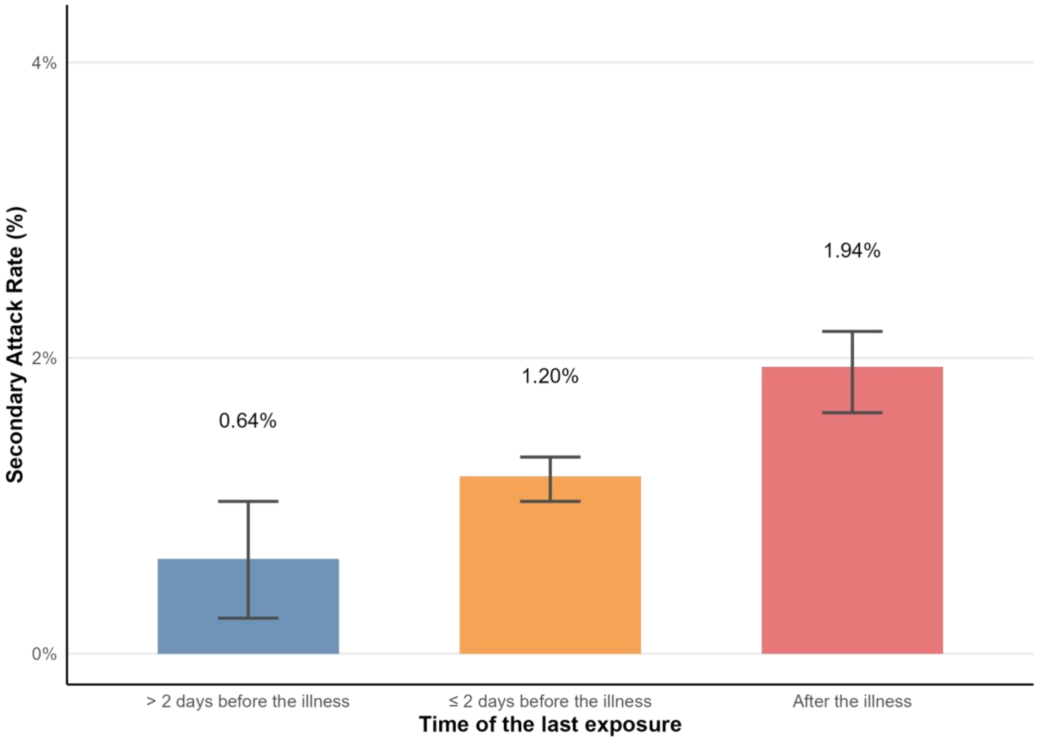

Supplementary Figure2. The secondary attack rates of SARS-CoV-2 among close contacts across different time of the last exposure.

Supplementary Table1. The distribution of Centralized quarantine duration for close contacts during 2020-2022， northeastern Shenzhen, China.

Duration of Centralized quarantine (days)	N (%)	Implement period
5	844 (2.7%)	2022
7	14188 (45.4%)	2022
10	5807 (18.6%)	2022
14	10411 (33.3%)	2020-2022
21	28 (0.1%)	2021

Supplementary Table2. Interactions between vaccination status and age in secondary infection of SARS-CoV-2 among close contact

Interactions between vaccination status and age	B	SD	P	OR (95% CI)
Unvaccinated*18-49 years				1[ref]
Partially vaccinated for over 6 months*<18 years	-0.729	0.849	0.391	0.48(0.07,2.19)
Partially vaccinated for less than 6 months*<18 years	1.281	1.498	0.393	3.6(0.13,101.57)
Fully vaccinated for over 6 months*<18 years	0.452	0.327	0.166	1.57(0.83,3.01)
Fully vaccinated for less than 6 months*<18 years	-0.605	0.886	0.495	0.55(0.07,2.82)
Booster vaccinated for over 6 months *<18 years	NA	NA	NA	NA
Booster vaccinated for less than 6 months *<18 years	NA	NA	NA	NA
Partially vaccinated for over 6 months*≥50 years	-0.716	1.108	0.518	0.49(0.03,3)
Partially vaccinated for less than 6 months*≥50 years	1.187	1.469	0.419	3.28(0.12,89.05)
Fully vaccinated for over 6 months*≥50 years	0.544	0.449	0.225	1.72(0.69,4.07)
Fully vaccinated for less than 6 months*≥50 years	-0.467	1.141	0.682	0.63(0.03,4.35)
Booster vaccinated for over 6 months *≥50 years	0.496	0.316	0.117	1.64(0.89,3.08)
Booster vaccinated for less than 6 months *≥50 years	0.718	0.545	0.188	2.05(0.66,5.76)
